# Immediate and Long-Term Effectiveness of Proprioceptive Neuromuscular Facilitation and Static Stretching on Joint Range of Motion, Flexibility, and Electromyographic Activity of Knee Muscles in Older Adults

**DOI:** 10.3390/jcm12072610

**Published:** 2023-03-30

**Authors:** Sahar Zaidi, Asfak Ahamad, Anam Fatima, Irshad Ahmad, Deepak Malhotra, Wafa Hashem Al Muslem, Sahar Abdulaziz, Shibili Nuhmani

**Affiliations:** 1 Centre for Physiotherapy and Rehabilitation Sciences, Jamia Millia Islamia, New Delhi 110025, India; 2 Department of Physiotherapy, Jamia Hamdard, New Delhi 110062, India; 3 Department of Physiotherapy, Faculty of Allied Health Sciences, Manav Rachna International Institute for Research and Studies, Faridabad 121004, India; 4 Department of Physical Therapy, College of Applied Medical Sciences, Imam Abdulrahman Bin Faisal University, Dammam 34212, Saudi Arabia

**Keywords:** contract–relax, electromyography, elders, flexibility, range of motion

## Abstract

**Introduction and Objective:** Previously, various stretching techniques were compared to study their effects on the different physiological parameters of hamstring muscles in the elderly population. There is no research that compares the immediate and long-term effects of proprioceptive neuromuscular facilitation-contract–relax (PNF-CR) and static stretching (SS) techniques on knee range of motion (ROM), hamstring flexibility, and knee flexor muscle EMG activity in the elderly. This study intends to compare the same. **Methods:** A total of 30 males aged 55–75 years were randomly assigned into the PNF-CR group (*n* = 10), SS group (*n* = 10), and control group (*n* = 10). The PNF-CR group received four trials of the contract–relax technique, the SS group received passive stretching of an 80 s duration by the therapist, and the control group received no intervention. A total of 12 sessions were given during the four-week period. Knee range of motion, electromyographic activity of the biceps femoris, and the sit-and-reach test were taken for the dominant side thrice: pre-intervention, immediately after stretching, and after the training period. **Results:** A statistically significant difference was observed in the maximum voluntary isometric contraction (MVIC) of biceps femoris between the PNF and the control groups (*p* = 0.01) after four weeks of intervention. The knee ROM and hamstring flexibility for the PNF group showed significant improvement immediately post-test (*p* = 0.01) and after four weeks of training (*p* = 0.07 and *p* = 0.001). SS showed significant results for both ROM and flexibility after four weeks of intervention (*p* = 0.001), and significant immediate post-test improvements were seen for ROM only (*p* = 0.007). **Conclusions:** PNF stretching has an immediate, as well as long-term, effect on knee ROM and hamstring flexibility, whereas it has only a long-term effect on muscle electromyographic activity. SS has an immediate, as well as long-term, effect on knee ROM and only a long-term effect on hamstring flexibility, without any immediate or long-term effects on muscle electromyographic activity.

## 1. Introduction

Aging can be defined as the gradual decline of the body’s ability to respond to its environment [1]. It has been seen that range of motion (ROM) decreases with increasing age. The biological changes associated with aging are related to the loss of joint ROM in individuals past the fourth decade of life [2,3]. These individuals also exhibit an increase in muscle stiffness and muscle atrophy, an increase in type I collagen, as well as the reorganization of the motor unit [2,3]. Alteration in ROM and electromyographic (EMG) activity in the older population can be presumed to be due to the aging process [4].

Different stretching techniques may have variable effects on ROM and EMG activity [2,5]. Proprioceptive neuromuscular facilitation (PNF) techniques make use of either proprioceptive stimulation for the strengthening (facilitation) or relaxation (inhibition) of muscle groups. One of the PNF principles states that voluntary muscular contractions are performed in combination with muscle stretching to reduce muscular contraction’s reflexive components, promote relaxation, and subsequently increase joint ROM. PNF stretch techniques have been demonstrated to increase joint ROM, compared to non-PNF stretch techniques [2]. These techniques are commonly used in athletic and clinical environments to enhance active and passive ROM and, in turn, optimize motor performance and rehabilitation [6,7].

PNF stretch techniques can be used to increase ROM in older adults as well. Older adults exhibit a response to PNF stretch techniques that is similar to the reaction of younger adults. In the latter, the agonist contract–relax (ACR) technique achieved greater knee joint ROM and muscle EMG activity, compared to the contract–relax (CR) and static stretch (SS) conditions [8] when comparing biceps femoris and gastrocnemius muscles.

One repetition of PNF stretching effectively improves acute hamstring extensibility in young adults. Additionally, in 2009, Higgs and Winter noted that three repetitions of PNF stretching three times a week was sufficient to produce meaningful improvements in flexibility for young collegiate-level athletes. Moreover, it has been postulated that PNF stretching significantly increases ROM and hamstring flexibility [9,10,11]. Consequently, significant work on this aspect proved that PNF stretch techniques are beneficial in improving hamstring muscle flexibility, decreasing the risk of injury, and increasing performance, EMG activity, muscle torque, and static balance [12,13,14,15].

Literature does not report any significant difference between static stretching and PNF stretching exercises when tested in young adults. Static stretching and PNF stretching both are effective in increasing hamstring flexibility [16] in young adults. In 2018, researchers confirmed the findings of a study conducted by Feland et al., 2001, which found that there were no differences between the PNF, static stretch, and control groups on older people [17]. However, as far as we know, there is no research that compares the effects of four weeks of PNF-CR and static stretching techniques on knee ROM, hamstring flexibility, and knee flexor muscle EMG activity in older people. Therefore, the purpose of this study is to compare the long-term effects of four weeks of the PNF-CR stretch technique with that of the static stretch technique on knee ROM, hamstring flexibility, and knee flexor muscle EMG activity in the older population.

## 2. Methods

### 2.1. Sample Size Calculation

The sample size was determined using the values of active knee ROM [2]. Ten subjects (including 20% dropouts) per group were necessary based on the effect size of 0.708 (calculated by Ferber et al., 2002) at an alpha level 0.05 and a power of 95%. Therefore, a total of 30 subjects were required for the study, with a dropout rate of 20%. This sample size was calculated using G Power 3.1.9.2 software.

### 2.2. Participants

A sample of convenience constituting 30 males was recruited based on the inclusion and exclusion criteria. Inclusion criteria were male adults aged between 55 and 75 years, able to do activities of daily living (ADL) without assistance, and able to comprehend and follow instructions. Exclusion criteria were Grade III or IV osteoarthritis or any other musculoskeletal condition affecting the muscle length, history of any surgery to the hip, knee, low back, or ankle, history of any medication (anti-inflammatory, for pain relief, or anti-arthritic) in the previous six months, history of life-threatening disease (neurological disease, cardiovascular disease, severe hypertension), and passive full-knee extension (popliteal angle 180 degrees). The purpose, methodology, potential risks, and their rights as a research subject were explained to the participants. Following which, the participants filled out an informed consent form. Participants who were willing to participate were recruited from the Out Patient Department, Center for Physiotherapy and Rehabilitation Sciences (CPRS), Jamia Millia Islamia (JMI), New Delhi, India.

### 2.3. Study Design

**Figure 1:**
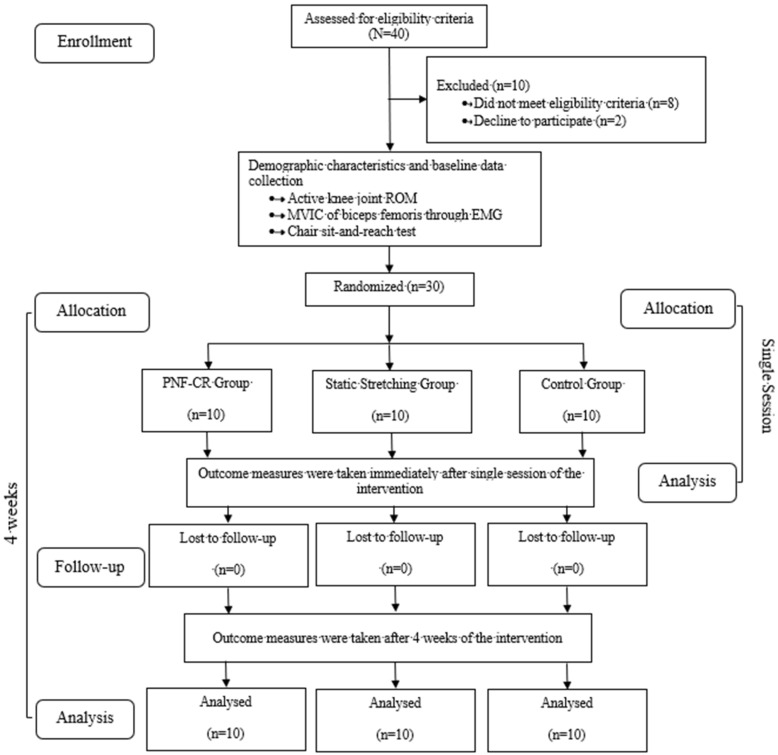
Flow chart of the patients who participated in the study (Consolidated Standards of Reporting Trials (CONSORT) diagram).

This was an outcome assessor-blinded pre-test–post-test randomized controlled trial, with two experimental groups {PNF-CR and static stretching (SS)} and a control group. The study was approved by the Institutional Review Board, Jamia Millia Islamia, New Delhi, India (Proposal no. 16/9/121/JMI/IEC/2017). The research was conducted in line with the principles declared by Helsinki and its amendments [18]. After fulfilling the eligibility criteria, the participants were randomly allocated into the 3 groups using block randomization with block size 3 and an allocation ratio of 1:1:1. Enrollment and assignment of participants were performed by an investigator who was neither a part of the assessment nor a part of the implementation of intervention. The study design is presented in Figure 1.

### 2.4. Procedure

All identifying information on the consent form and demographic data were kept confidential by assigning a number to each subject of this study. All participants underwent a familiarization session of the intervention for a week, incorporating three sessions. It was necessary to use proper technique and reduce risk of injury during the actual implementation of the intervention. Participants were given 24 to 48 h of rest (after familiarization), and then baseline assessment was taken for the maximal voluntary isometric contraction (MVIC) of the biceps femoris, the active ROM of knee, and the chair sit-and-reach test. Participants allocated to the PNF-CR group received the contract–relax technique of proprioceptive neuromuscular facilitation stretching, the SS group received passive stretching from the therapist, and the control group received no intervention. A total of twelve sessions (three sessions per week) were given during the four-week period for both the PNF-CR and SS groups. Post-assessment (immediate) results were taken after the first training session. Additionally, at the end of four weeks of intervention, participants were assessed again for the same variables. Participants allocated to intervention groups were treated in the Out Patient Department, CPRS, JMI, New Delhi, India.

### 2.5. Intervention

#### 2.5.1. PNF-CR Group

The subjects were made to lie down in the supine position. The non-dominant thigh was strapped in at zero degrees of hip flexion and the dominant thigh in at 90° of hip flexion. The intervention began with the examiner passively extending the subject’s knee to the point of muscular restriction. When this position was attained, the subject was instructed to flex the knee with maximal force (isometric knee flexor contraction) against the examiner’s resistance for five seconds. Then, the subject was taught to relax the knee musculature completely. Simultaneously, the examiner passively extended the knee to a newly attained point of muscular restriction and maintained the position for five seconds [2]. The procedure was repeated. One trial had two contractions, each followed by five seconds of muscle stretching, and there was a total of four trials.

#### 2.5.2. Static Stretching Group

The subjects were made to lie down in the supine position. The non-dominant thigh was strapped in at zero degrees of hip flexion, and the dominant thigh was strapped in at 90° of hip flexion. The subjects were asked to concentrate on relaxing the muscles of the legs and thighs as much as possible. At the same time, the experimenter passively extended the knee joints to the point of muscular restriction. Knee extensions were gradually increased manually and maintained for 80 s [2,8].

### 2.6. Outcome Measures

#### 2.6.1. Maximal Voluntary Isometric Contraction for Biceps Femoris

Surface EMG was used to record the MVIC of biceps femoris. The disposable bipolar silver/silver chloride surface electrodes, with 20 mm of inter-electrode distance, were placed parallel to the muscle fibers, overlying the muscle belly of the biceps femoris muscle, according to the SENIAM recommendations. To achieve an optimal EMG signal and low impedance, a four cm^2^ skin area was shaved off and cleaned. The MVIC of the biceps femoris was performed. The subjects were asked to lie prone with their hips straight, knee flexed at 90°, and pelvis stabilized with a strap [2]. Then, the subjects were instructed to maximally flex their knee for five seconds against resistance by the investigator, which was applied just above the ankle joint posteriorly.

#### 2.6.2. Active Knee Joint ROM Measurement

Active knee ROM was assessed using a universal goniometer. The subject was asked to lie supine. The investigator palpated the greater trochanter, the lateral epicondyle of the femur, and the lateral malleolus of the fibula. One horizontal line was drawn with the femur’s lateral epicondyle as reference, while the second horizontal line was marked with the lateral malleolus of the fibula. The non-dominant thigh was strapped in at zero degrees of hip flexion and the dominant thigh at 90° of hip flexion. The goniometer’s fulcrum was placed on the lateral epicondyle of the femur, the stationary arm reference with greater trochanter, and the moveable arm reference with the lateral malleolus of the fibula. Then, the subject was asked to straighten the knee as much as possible. Hence, the active knee extension ROM was recorded. Subjects were also advised to keep their low back flat on the table during the procedure to limit further possible pelvic rotation during the measurement [2,8]. The standard error of measurement reported for the universal goniometric measurement of knee extensions ranged from 0.72° to 4.2°, with most of the studies reporting it to be around 1.5° ± 0.5° [19,20,21].

#### 2.6.3. Chair Sit-and-Reach Test (CART)

CART was used to assess the hamstring muscle’s length flexibility. The subjects were seated in a chair (17 in high seat) and were asked to move forward until they sat near the front edge. The chair was placed against a wall and checked to see that it would remain stable throughout the testing. Subjects were asked to extend their preferred leg in front of their hip, with the heel on the floor and the foot in dorsiflexion (at approximately a 90° angle), and to bend the other leg so that the sole was flat on the floor, about 6–12 inches to the side of the body’s midline. With the extended leg as straight as possible, with hands on top of each other and with palms down (tips of the middle fingers even), subjects slowly bent forward at the hip joint, keeping the head and spine as straight as possible. Subjects were instructed to reach down the extended leg to touch the tip of the middle toes. The subjects held a brief static position (for 2 s) while the administrator recorded the “reached score” using an 18-inch ruler positioned parallel to the lower leg (shin). The middle of the toe at the end of the shoe represented a “zero” score. If the patient reached short of the toes, it was recorded as a minus score, and if the patient reached beyond the toes, it was recorded as a plus score [22].

### 2.7. Data Analysis

Data were analyzed with SPSS Version 21.0 (IBM Co., Armonk, NY, USA). The demographic characteristics and the baseline (pre-treatment) criterion measures were compared between the three groups using one-way ANOVA. A 3 × 3 split-plot repeated measure ANOVA was used to find out the group effect (PNF-CR, static stretching, and control group), time effect (pre-intervention, post (immediate)-intervention, and post (four week)-intervention), and group × time interaction effect. If the group effect, time effect, or interaction effect was found to be significant, then the post hoc pairwise comparison was analyzed using the Bonferroni correction test. Additionally, the one-way ANOVA was used between the three groups at the post-immediate and post-four-week interventions. The significance level was set at *p* < 0.05, and the confidence interval was set at 95%. Data were presented as mean ± SD unless otherwise indicated.

## 3. Result

All the groups were similar at the baseline for all outcome measures and demographic variables (*p* > 0.05). 

Demographic characteristics, such as age, weight, height, and body mass index (BMI), were found to be non-significant between the groups. MVIC, ROM, and CART scores were found to be statistically non-significant between the three groups at baseline (Table 1).

### 3.1. EMG Activity of Biceps Femoris Muscle

The MVIC of biceps femoris showed a statistically significant time effect (F(2,54) = 195.32, *p* < 0.001) and group × time interaction (F(4,54) = 57.49, *p* < 0.001), whereas no significant difference was found in the group effect (F(2,54) = 1.16, *p* = 0.209) (Table 2). A post hoc pairwise comparison indicated that there was a statistically significant difference in the MVIC activity of the biceps femoris muscle when comparing pre-intervention and post-immediate intervention (*p* < 0.001) results, as well as pre-intervention and post-four-week intervention results (*p* < 0.001) (Table 3). Only the PNF-CR and control groups (*p* = 0.014) showed significant differences after four weeks, whereas no statistically significant differences in the MVIC were found between other groups during the post-immediate, as well as post-four-week, intervention (Table 4).

### 3.2. Active Knee Joint ROM

Knee ROM showed statistically significant time effect (F(2,54) = 799.77, *p* < 0.001), group effect (F(2,54) = 15.56, *p* < 0.001), and group × time interaction (F(4,54) = 173.04, *p* < 0.001) (Table 2). A post hoc pairwise comparison indicated that there was a significant difference in the knee ROM when comparing pre-intervention and post-immediate intervention (*p* < 0.001), as well as pre-intervention and post-four-week intervention (*p* < 0.001) (Table 3). Additionally, there was a statistically significant difference in the PNF-CR and static stretching group when compared with the control group at the post-immediate (*p* < 0.001 and *p* = 0.007 respectively), as well as the post-four-week, intervention (*p* < 0.001 and *p* < 0.001 respectively) (Table 4). No difference was found between PNF-CR and static stretching group at the post-immediate, as well as the post-four-week, intervention (Table 4).

### 3.3. Chair Sit-and-Reach Test (CART)

**Figure 2:**
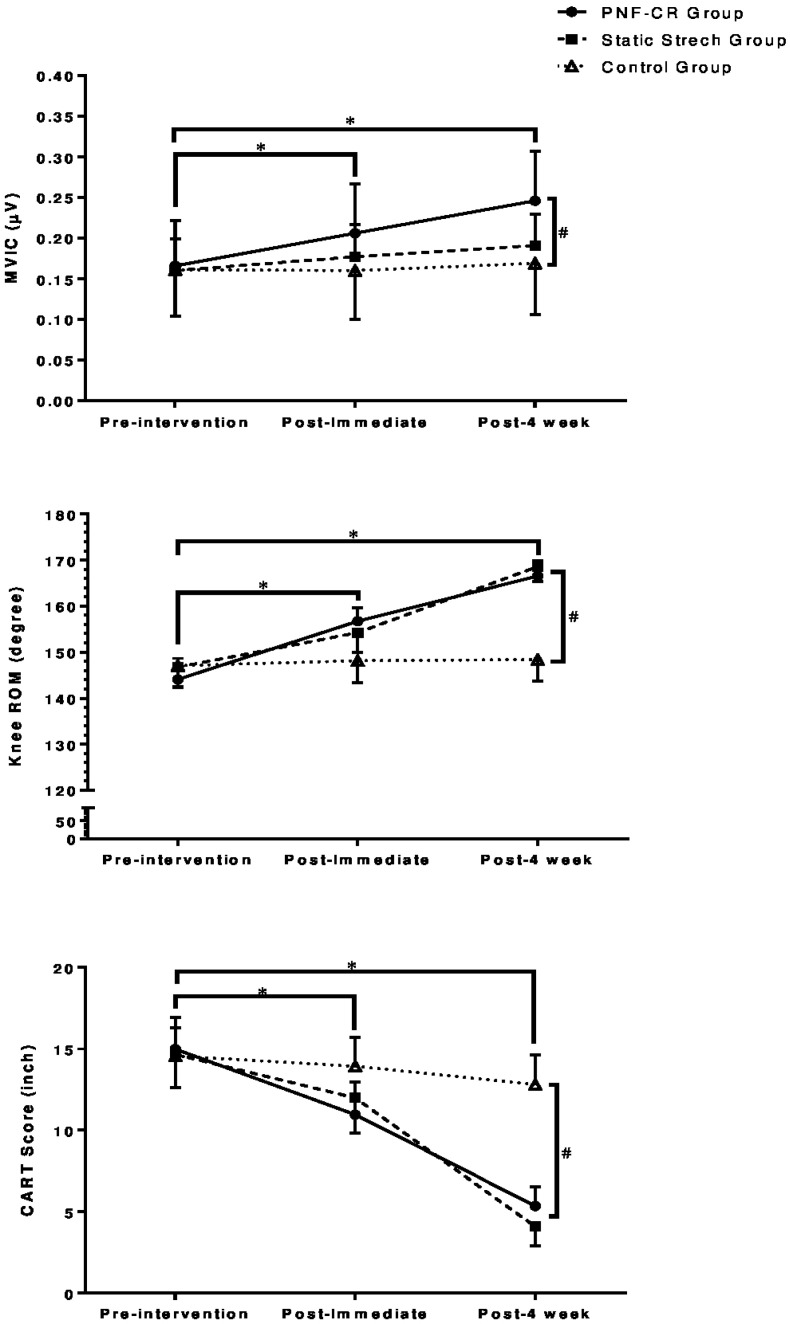
Outcome measures at baseline, immediately after intervention, and after 4 weeks of intervention for the PNF-CR, static stretching, and control groups. *: significant difference between different time points; #: significant difference between the groups.

The CART score showed statistically significant time effect (F(2,54) = 656.75, *p* < 0.001), group effect (F(2,54) = 14, *p* < 0.001) and group × time interaction (F(4,54) = 100.52, *p* < 0.001) (Table 2). Post hoc pairwise comparison indicated that there was a significant difference in the CART score when comparing pre-intervention and post-immediate intervention (*p* < 0.001), as well as pre-intervention and post-four-week intervention (*p* < 0.001) (Table 3). The CART score showed a significant difference between the PNF-CR group and the control group at the post-immediate (*p* = 0.007) and post-four-week interventions (*p* < 0.001), whereas the static stretching group and the control group showed a significant difference only at the post-four-week intervention (*p* < 0.001) (Table 4). Meanwhile, no difference was found between the PNF-CR group and the static stretching group at the post-immediate and post-four-week interventions (Table 4) (Figure 2).

## 4. Discussion

The purpose of this study was to compare the effectiveness of PNF-CR and SS immediately after as well as post-four-week intervention between three groups in improving hamstring muscle flexibility, as measured by ROM, CART, and EMG activity through the MVIC of the biceps femoris muscle. Our study found that both techniques (PNF-CR and SS) were effective at improving the knee ROM immediately and after four weeks of intervention. The biceps femoris muscle activity (MVIC) responded to the PNF-CR technique only after the four-week intervention. We also found that PNF-CR had a significant effect on hamstring flexibility after a single intervention, and the effect was further enhanced after a structured training program of four weeks. SS had only a long-term effect on hamstring flexibility without any immediate or long-term effects on the muscle’s electromyographic activity. The absolute long-term effect of SS on hamstring flexibility was comparatively greater than that of the PNF-CR technique, with both being statistically significant.

In line with previous research, Konrad et al. conducted a study on 122 subjects to assess the effects of various stretching techniques, i.e., static stretching, ballistic stretching, and PNF-CR, on knee ROM; they did not find any statistically significant differences between these stretching technique groups (*p* > 0.05) [23]. Additionally, Puentedura et al. compared the effect of PNF-hold–relax and static stretching techniques on the active knee extension ROM; they reported no significant difference between these two stretching techniques on the active knee joint extension ROM (*p* = 0.782) [24].

Additionally, Funk et al. analyzed PNF stretching with the addition of two exercises (cycling and weight lifting) and reported that it was more effective at increasing hamstring flexibility when compared with PNF stretching without exercise or static stretching counterparts. They also reported that there was no significant difference in knee ROM between the PNF and static stretching groups [25].

Feland et al. conducted a study on 97 subjects to compare the effects of two types of stretching (PNF-CR and static stretching) on older adults’ hamstring flexibility. They concluded that there was no significant difference between the PNF-CR and static stretching groups (*p* = 0.1461) [8]. Furthermore, it was stated there was no significant difference between the PNF-hold–relax stretching and SS’s effect on knee ROM, but MVIC significantly increased only in the static stretching group when compared to that in the PNF-hold–relax stretching group [26]. Later, a study reported that PNF stretching significantly decreased the MVIC EMG activity of biceps femoris in comparison to static stretching [23].

According to Ferber et al., the use of the ACR PNF technique on hamstring muscles in older adults was more beneficial for increasing knee joint ROM and EMG activity than the PNF-CR technique and the static stretching technique [2].

Our study demonstrated a significant effect of PNF-CR stretch techniques on increased EMG activity during MVIC after the four-week intervention, compared with that in the control group. Nevertheless, static stretching, both immediately and after four weeks of intervention, did not result in any significant improvement in EMG activity during MVIC.

Several researchers reported no significant differences between the PNF technique and static stretching techniques in knee ROM. Beltro et al. found that no significant difference occurred with either short-term or long-term PNF and static stretching stretch techniques in the active knee extension ROM, respectively. Although there was no significant difference, the percentage improvements in the ROM following the long-term intervention were comparatively more than following the short-term intervention (*p* = 0.001) [27].

According to Minshull et al., the flexibility training of 8 weeks with PNF and passive stretching techniques improved hip flexibility [28]. In 2009, Yuktasir and Kaya demonstrated that after a six-week training period using static stretching and the PNF, respectively, in both groups, there was a significant increase in knee extension ROM when compared to the control group (*p* < 0.001). They also reported no significant difference between the static stretching and PNF groups.

Davis et al. stated that there was a significant (group x time) interaction between stretching technique and time (*p* < 0.0016). At the baseline, there was no significant difference in hamstring flexibility between groups. At two weeks, flexibility significantly increased in the static stretching group (*p* ≤ 0.05). At 4 weeks, all three stretching groups had considerably increased flexibility from baseline (*p* ≤ 0.05). Static stretching was the only technique that significantly increased flexibility, compared with the control group (*p* ≤ 0.05) [29].

According to the findings of the study of Shadmehar et al., after ten sessions of static stretching and PNF stretching exercises in two groups, respectively, there was no significant difference in knee extension ROM for both groups, compared to the baseline measurements. There were no significant differences between groups in knee extension ROM [30].

Recently, Lempke et al. conducted a study on PNF stretching exercise effectiveness versus static stretching exercise on hip joint flexion ROM [17]. There was no significant difference between the active straight leg raise, active knee extension angle, and active knee extension test between the PNF and static stretching techniques [17]. Hill et al. compared PNF stretching programs’ immediate effects with passive stretching programs for hamstring flexibility; they concluded that there was no significant difference in the active knee extension test and the active knee extension angle (hamstring flexibility) between groups after the PNF and static stretching techniques, respectively [31]. Our study demonstrated that three repetitions of PNF-CR and static stretching are more effective for immediate effects of hamstring flexibility and improving EMG activity during MVIC; however, there were no differences between the two techniques. At four weeks after intervention, the results demonstrated that both techniques were more beneficial for increasing muscle flexibility in older people, but between groups, PNF-CR and static stretching strength significantly increased only in PNF-CR.

The limitations of this study are that it was performed on a limited number of subjects from a single institution. This study was conducted on older people only; thus, results cannot be generalized. It is advised to use an electrogoniometer for the accurate and precise measurement of the range of motion. Since the patient population in this study was healthy older adults, its findings cannot be used to design a treatment protocol for older adults with comorbidities. Additionally, the EMG activity of only the biceps femoris muscle was evaluated, and the other muscles, such as semimembranosus and semitendinosus, were not evaluated.

The present study’s strengths are that this was the first study that compared PNF-CR and static stretching techniques with a control group to determine the best method for immediate and long-term effects on hamstring muscle flexibility in older people. In previous studies, a temporary hold–relax technique or soft tissue mobilization to maintain the knee ROM was used; however, in this study, exercises were used as interventions to increase the ROM to retain the muscle hamstring flexibility [24,32,33]. This could be justified as a better and long-term solution in the elderly population.

In the future, studies that use more extended follow-up periods are recommended in order to see the intervention effect. Further, there is a need to determine the hamstring muscle’s activation during sit-to-stand, stair ascending, and stair descending maneuvers.

### Clinical Implications

As mentioned above, a number of physiological changes occur as a consequence of aging. Decreased flexibility of the musculotendinous units and decreased muscle strength are a couple of them, which may have major direct or indirect impacts on the movement biomechanics and qualities of life of the elderly population. Increased stiffness of the muscles will lead to reductions in their force production. This reduction can be offset by the application of the PNF-CR technique. The same was demonstrated by the significant improvement of the MVIC after the long-term application of PNF-CR. Therefore, the present study’s findings will be instrumental when making treatment protocols for older adults to improve hamstring flexibility and knee ROM, which will, in turn, improve their activities of daily living and overall quality of life by reducing the possibility of ailments and future injuries prevalent in this population due to tight musculature. It was demonstrated that PNF-CR would have an added advantage of improvement in strength along with flexibility because of the isometric activation of the muscles during the procedure. Both PNF-CR and SS techniques do not require any specialized equipment, such as IASTM tools or foam rollers, for implementation and are relatively easy to apply in clinical as well outdoor settings, such as community participation workshops or training sessions for the elderly.

## 5. Conclusions

The present study results suggest that the PNF-CR stretching technique is better for immediate effects on active knee ROM and hamstring muscle flexibility and for long-term effects on hamstring muscle activity and flexibility. The static stretching is better for long-term effects of flexibility only.

## Figures and Tables

**Table 1:** Demographic characteristics and outcome variables at baseline for PNF-CR, static stretching, and control groups.

Variables	PNF-CR Group (*n* = 10)	Static Stretching Group (*n* = 10)	Control Group (*n* = 10)	One-Way ANOVA
	Mean ± SD	Mean ± SD	Mean ± SD	*p*-Value
Age	59.20 ± 3.15	59 ± 3.83	58.7 ± 3.02	0.94
Weight	67.10 ± 6.35	68.6 ± 3.53	68.5 ± 4.9	0.76
Height	1.64 ± 0.08	1.67 ± 0.46	1.67 ± 0.07	0.52
BMI	24.88 ± 2.37	24.37 ± 1.01	24.56 ± 2.03	0.82
MVIC	0.16 ± 0.56	0.16 ± 0.04	0.161 ± 0.05	0.96
Knee ROM	146.1 ± 4.57	146.73 ± 4.2	147.06 ± 4.72	0.88
CART	14.98 ± 1.94	14.66 ± 2.03	14.56 ± 1.72	0.87

PNF-CR: proprioceptive neuromuscular facilitation-contract–relax; BMI: body mass index; MVIC: maximal voluntary isometric contraction; ROM: range of motion; CART: chair sit-and-reach test.

**Table 2:** Outcome measures at baseline, immediately after intervention, and after 4 weeks of intervention for PNF-CR, static stretching, and control groups.

Variables	PNF-CR Group (*n* = 10)	Static Stretching Group (*n* = 10)	Control Group (*n* = 10)	Time (T) Effect	Group (G) Effect	G × T Interaction
	Mean ± SD	Mean ± SD	Mean ± SD	η_p_^2^ (*p*-Value)	η_p_^2^ (*p*-Value)	η_p_^2^ (*p*-Value)
MVIC				0.87 (<0.001) *	0.11 (0.209)	0.81 (<0.001) *
Pre	0.16 ± 0.05	0.16 ± 0.04	0.16 ± 0.05			
Post (Immediate)	0.21 ± 0.06	0.17 ± 0.04	0.16 ± 0.06			
Post (4 weeks)	0.24 ± 0.06	0.19 ± 0.03	0.17 ± 0.06			
ROM				0.96 (<0.001) *	0.53 (<0.001) *	0.92 (<0.001) *
Pre	144.1 ± 4.57	146.73 ± 4.2	147.06 ± 4.72			
Post (Immediate)	156.7 ± 3	154.23 ± 4.19	148.16 ± 4.77			
Post (4 weeks)	166.53 ± 3.31	168.60 ± 3.31	148.40 ± 4.71			
CART				0.96 (<0.001) *	0.51 (<0.001) *	0.88 (<0.001) *
Pre	14.98 ± 1.94	14.66 ± 2.03	14.56 ± 1.72			
Post (Immediate)	10.96 ± 1.98	11.98 ± 2.16	13.93 ± 1.79			
Post (4 weeks)	5.35 ± 1.18	4.10 ± 1.22	12.81 ± 1.83			

PNF-CR: proprioceptive neuromuscular facilitation-contract–relax; MVIC: maximal voluntary isometric contraction; ROM: range of motion; CART: chair sit-and-reach test; *: significant difference.

**Table 3:** Post hoc pairwise comparison using the Bonferroni correction between pre- and post (immediate)-intervention and pre- and post (4 weeks)-intervention for all groups combined.

Variables	Pre vs. Post (Immediate)	Pre vs. Post (4 Weeks)
MVIC	<0.001 *	<0.001 *
ROM	<0.001 *	<0.001 *
CART	<0.001 *	<0.001 *

MVIC: maximal voluntary isometric contraction; ROM: range of motion; CART: chair sit-and-reach test; *: significant difference.

**Table 4:** Post hoc pairwise comparison using Bonferroni correction between the three groups at different time points, i.e., pre- and post (immediate)-intervention and post (4 weeks)-intervention.

Variables	PNF-CR vs. Control	Static Stretch vs. Control	PNF-CR vs. Static Stretch
MVIC			
Pre	-	-	-
Post (Immediate)	0.209	1	0.711
Post (4 weeks)	0.014 *	1	0.107
ROM			
Pre	-	-	-
Post (Immediate)	<0.001 *	0.007 *	0.557
Post (4 weeks)	<0.001 *	<0.001 *	0.718
CART			
Pre	-	-	-
Post (Immediate)	0.007 *	0.112	0.79
Post (4 weeks)	<0.001 *	<0.001 *	0.193

PNF-CR: proprioceptive neuromuscular facilitation-contract–relax; MVIC: maximal voluntary isometric contraction; ROM: range of motion; CART: chair sit-and-reach test; *: significant difference.

## Data Availability

The data set for the result of this study will be available from the corresponding author upon reasonable request.
